# Evaluation of the Efficacy of COVID-19 Booster Vaccinations in Healthcare Personnel

**DOI:** 10.3390/vaccines10111797

**Published:** 2022-10-26

**Authors:** Chung-Jong Kim, Ji-Yun Bae, Kang-Il Jun, Jihee Kim, Hee-Jung Son, Hae-Sun Chung, Soo-Kyung Kim, Soohyun Kim, Dohsik Minn, Hee-Jung Choi

**Affiliations:** 1 Department of Internal Medicine, Ewha Womans University Seoul Hospital, Seoul 07804, Korea; 2 Ewha Education and Research Center for Infection, Seoul 07985, Korea; 3 Office of Infection Control, Ewha Womans University Seoul Hospital, Seoul 07804, Korea; 4 Department of Internal Medicine, Ewha Womans University Mokdong Hospital, Seoul 07985, Korea; 5 Office of Infection Control, Ewha Womans University Mokdong Hospital, Seoul 07985, Korea; 6 Department of Laboratory Medicine, Ewha Womans University Seoul Hospital, Seoul 07804, Korea; 7 Department of Laboratory Medicine, Ewha Womans University Mokdong Hospital, Seoul 07985, Korea; 8 Department of Diagnostic Immunology, Seegene Medical Foundation, Seoul 04805, Korea

**Keywords:** COVID-19 vaccines, COVID-19 vaccine booster shot, antibodies, SARS-CoV-2

## Abstract

This study aimed to investigate the efficacy of different COVID-19 booster vaccines by measuring the serum antibody titer. SARS-CoV-2 anti-nucleocapsid protein antibody (N-Ab), anti-spike protein antibody (S-Ab), and neutralizing antibody (Neut.Ab) were measured before and 4–6 weeks after booster vaccinations in healthcare personnel with a previous vaccination within 3–6 months. Personnel who previously received two doses of ChAdOx1 vaccine or two doses of BNT162b2 vaccine received the BNT162b2 vaccine (AAP and PPP groups, respectively). Personnel who previously received two doses of mRNA-1273 received the same vaccine as a booster dose (MMM group). Of the 917 participants, the AAP, MMM, and PPP groups comprised 837 (91.3%), 27 (2.9%), and 53 (5.8%) participants, respectively. The pre-booster S-Ab and Neut.Ab titer were significantly lower in the AAP group. After the booster vaccination, all participants were positive for S-Ab and Neut.Ab; furthermore, the S-Ab and Neut.Ab titer significantly increased in all three groups, although the post-booster S-Ab was lower in the AAP group than in the other groups. The post-booster Neut.Ab titer showed no significant difference among the groups. Our study’s results suggest that booster vaccination, after two prior vaccinations, shows a significant effect regardless of the type of vaccine administered.

## 1. Introduction

Coronavirus disease-19 (COVID-19) is an infectious disease that occurred at the end of 2019 and quickly spread worldwide in a short period of time, causing a pandemic [1]. Because of the spread of COVID-19, many countries have imposed lockdowns and travel restrictions, which have had a huge social and economic ripple effect [2,3]. A new antiviral treatment is being introduced and various treatments are being studied [4,5,6]. However, newly detected variants, such as omicrons, make this situation difficult to control. The high transmission rate of the newly developed variants of concern and the resulting large number of patients place an additional burden on medical resources. Therefore, the most important factor in the response to COVID-19 is massive immunization by vaccination [3].

The immunogenicity of the current WHO-approved COVID-19 vaccines is well known [7,8,9,10,11]. In particular, three types of vaccines, ChAdOx1, BNT162b2, and mRNA-1273, have been developed in many countries during the early stages of the COVID-19 outbreak and have proven their efficacy.

The recent prevalence of delta and omicron variants and immune waning [12,13,14] in vaccinated individuals overlap, leading to a global epidemic. Several countries are trying to solve this problem through booster vaccinations.

In this study, we aimed to investigate the response to booster vaccinations in participants who had received ChAdOx1, BNT162b2, or mRNA-1273 in the previous 3–6 months and to compare the antibody titers before and after booster vaccination to confirm the efficacy of the booster vaccination strategy.

## 2. Materials and Methods

### 2.1. Patients and Materials

This study was conducted with healthcare personnel (HCP) working in two university hospitals. They had been previously vaccinated with one of the following vaccines according to the policy of the Korean government: BNT162b2, ChAdOx1, or mRNA-1273. Only those who had previously been vaccinated according to the schedule of each vaccine were included in this study. There were no demographic differences between the indications for BNT162b2 and ChAdOx1 vaccination, but in the case of BNT162b2, HCP treating patients with severe COVID-19 were targeted with this vaccine, and ChAdOx1 was targeted for all other HCP. The indication for the mRNA-1273 vaccine is HCP who were 18–30 years of age, because ChAdOx1 use in these age groups was banned owing to the possibility of vaccine-induced immune thrombotic thrombocytopenia; the indication for ChAdOx1 vaccination was revised to suitable for use in people aged ≥30 years.

HCP who received two homologous prime-boost doses of BNT162b2 or ChAdOx1 were boosted with BNT162b2, and HCP who were homologous prime-boosted vaccinated with mRNA-1273 were boosted with a half dose of the primary mRNA-1273 vaccine, as authorized. In all HCP, booster vaccination was administered at least 3–6 months after the previous vaccination.

### 2.2. Study Protocol

This study was conducted only on personnel who agreed to participate in the study. Written informed consent was obtained before enrolment in the study. Upon consent to participate in the study, a pre-booster antibody test was performed 1–7 days before booster vaccination. Post-booster vaccination antibody titer tests were performed 4–6 weeks after booster vaccination. The patients were divided into three groups according to their vaccination history. Homologous prime-boosts vaccinated with BNT162b2 or ChadOx1 were grouped into PPP and AAP groups, respectively. Homologous prime-boosts vaccinated with mRNA-1273 were grouped into the MMM group.

### 2.3. Laboratory Protocol

Blood was collected from the participants and sera were separated. The serum samples were tested with an Elecsys Anti-SARS-CoV-2 assay (Roche Diagnostics, Mannheim, Germany), a quantitative Elecsys Anti-SARS-CoV-2 S assay (Roche Diagnostics) using a Cobas 8000 e801 unit (Roche Diagnostics), and neutralizing antibody (Neut.Ab) with R-FIND SARS-CoV-2 Neutralizing Antibody ELISA (SG Medical, Seoul, Korea), according to each manufacturer’s protocol.

The past COVID-19 infection history was confirmed with an Elecsys Anti-SARS-CoV-2 assay (Roche Diagnostics, Mannheim, Germany), which uses a recombinant protein representing the nucleocapsid antigen to detect the antibodies against SARS-CoV-2 nucleocapsid protein (N-Ab). The nucleocapsid antigen is not a target of those vaccines used in this study; therefore, it is a useful marker to determine whether the participant had been infected with SARS-CoV-2 in the past, regardless of COVID-19 vaccination.

To examine the humoral immunity response of the vaccine, quantitative Elecsys Anti-SARS-CoV-2 S assay (Roche Diagnostics) was performed. This assay uses a recombinant protein representing the receptor-binding domain (RBD) of the spike antigen, which favors quantitative determination of high-affinity antibodies against SARS-CoV-2 spike protein (S-Ab). S-Ab ≥ 0.80 U/mL and N-Ab COI ≥ 1.0 were considered positive.

The R-FIND SARS-CoV-2 neutralizing antibody ELISA detects Neut.Ab using a competitive ELISA format. For the R-FIND SARS-CoV-2 neutralizing antibody ELISA, the sample was pre-mixed with the RBD-horseradish peroxidase (HRP) solution in parallel with the control. The mixture was added to a microwell strip pre-coated with ACE2 protein. RBD-HRP and ACE2 protein interactions were inhibited by SARS-CoV-2 Neut.Ab against RBD. The Neut.Ab unbound to RBD-HRP and RBD-HRP bound to the non-neutralizing antibody were captured on the plate. After washing, the HRP substrate solution was added and the color change was read using a microplate reader. Signal inhibition was calculated as (1—optical density of sample/average optical density of negative control) × 100. A signal inhibition of ≥30% was considered positive.

### 2.4. Data Collection

The baseline characteristics of the enrolled participants, such as age, sex, previous COVID-19 infection history, and type and time of previous COVID-19 vaccination, were investigated.

### 2.5. Statistical Analysis

Student’s *t*-test or chi-squared test were used to compare the baseline characteristics between the groups. The antibody titer of S-Ab was compared using the geometric mean and the Neut.Ab titer was compared using the arithmetic mean. Student’s *t*-test was used for statistical comparisons. Statistical significance was set at *p* < 0.05.

## 3. Results

### 3.1. Baseline Characteristics

In total, 917 participants were enrolled in this study. There were 837 (91.3%) participants in the AAP group, 27 (2.9%) participants in the MMM group, and 53 (5.8%) participants in the PPP group. There were 172 (18.8%) men and 745 (81.2%) women. The interval between the second vaccine dose to pre-booster Ab tests was 188 ± 7, 140 ± 1, and 209 ± 26 days in the AAP, MMM, and PPP groups, respectively. The interval between the booster vaccination and post-booster Ab tests was 35 ± 5, 34 ± 3, and 34 ± 4 days in the AAP, MMM, and PPP groups, respectively. Seven participants with positive N-Ab results in the pre-booster test were excluded from subsequent analyses. Table 1 shows the baseline characteristics of each group.

### 3.2. Results of Antibody Analysis

All participants were positive for S-Ab before booster vaccination. Pre-booster Neut.Ab were positive in 560 participants (67.5%) in the AAP group, 27 participants (100%) in the MMM group, and 52 (98.1%) participants in the PPP group. Post-booster tests were performed in 677 participants (80.9%) in the AAP group, 20 (74.1%) participants in the MMM group, and 46 (86.8%) participants in the PPP group. One participant in the AAP group whose N-Ab was converted to positive in the post-booster test was excluded from the subsequent analysis. S-Ab and Neut.Ab were positive in all participants at the time of the post-booster test.

In the AAP group, the S-Ab titer before booster vaccination was 226.1 (±2.3) and the S-Ab titer after booster vaccination was 13,105.7 (±1.7) (*p* < 0.001). In the MMM group, the S-Ab titer before booster vaccination was 1723.8 (±1.6) and the S-Ab titer after booster vaccination was 19,932.3 (±1.5) (*p* < 0.001). In the PPP group, the S-Ab titer before booster vaccination was 712.0 (±2.2) and the S-Ab titer after booster vaccination was 16,360.1 (±1.7) (*p* < 0.001).

In the AAP group, Neut.Ab inhibition% was 44.0% (±23.6) before booster vaccination and 97.0% (±2.4%) after booster vaccination (*p* < 0.001). In the MMM group, before booster vaccination, Neut.Ab inhibition% was 91.6% (±3.9) and, after booster vaccination, Neut.Ab inhibition% was 97.2% (±0.6) (*p* < 0.001). In the PPP group, before booster vaccination, Neut.Ab inhibition% was 76.6% (±17.8) and, after booster vaccination, it was 97.5% (±0.6) (*p* < 0.001).

The S-Ab titer before booster vaccination was significantly lower in the AAP group and there was a significant difference among the three groups (226.1 vs. 1723.8 vs. 712.0, *p* < 0.001). After booster vaccination, the S-Ab titer was still significantly lower in the AAP group (13105.7 vs. 19932.3 vs. 16360.1, *p* < 0.001), but there was no significant difference between the PPP and MMM groups (*p* = 0.370).

**Figure 1:**
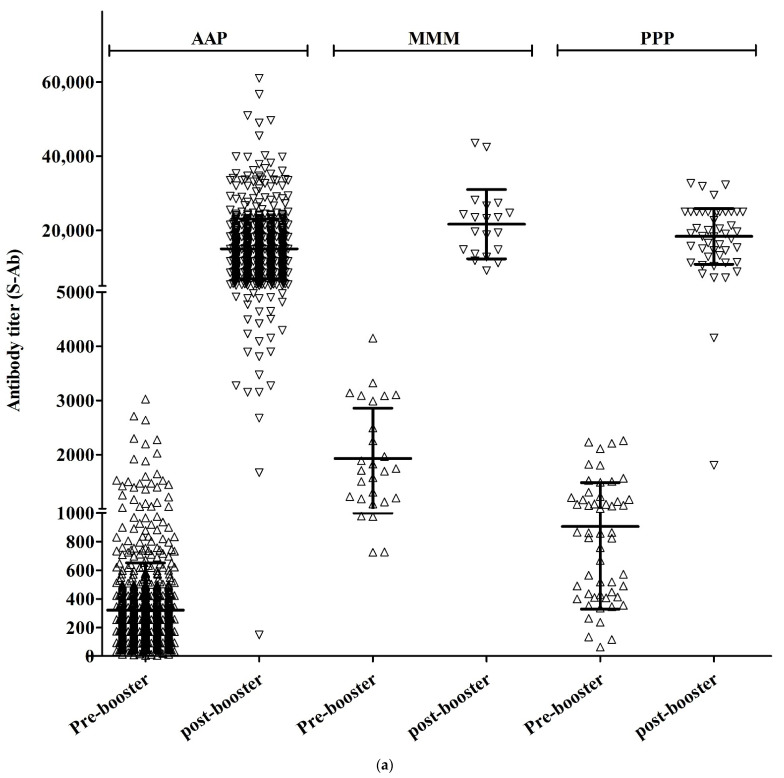

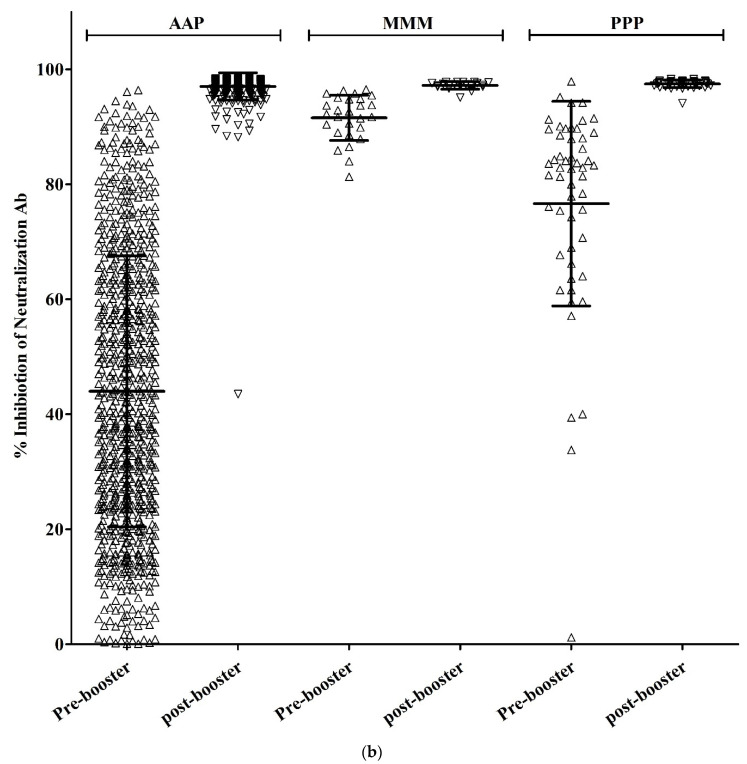
S-Ab (**a**) and Neut.Ab (**b**) titer before and after the COVID-19 booster vaccination in different vaccination groups.

Neut.Ab titer before booster vaccination was significantly lower in the AAP group (*p* < 0.001), but Neut.Ab titer after booster vaccination was not significantly different among the groups (*p* = 0.404) (Table 1, Figure 1).

### 3.3. Results of Antibody Analysis by Subgroup

A total of 514 participants (56.1%) were aged <40 years, with 63 males (12.3%) and 456 females (87.7%). There were 452, 25, and 37 participants in the AAP, MMM, and PPP groups, respectively.

**Figure 2:**
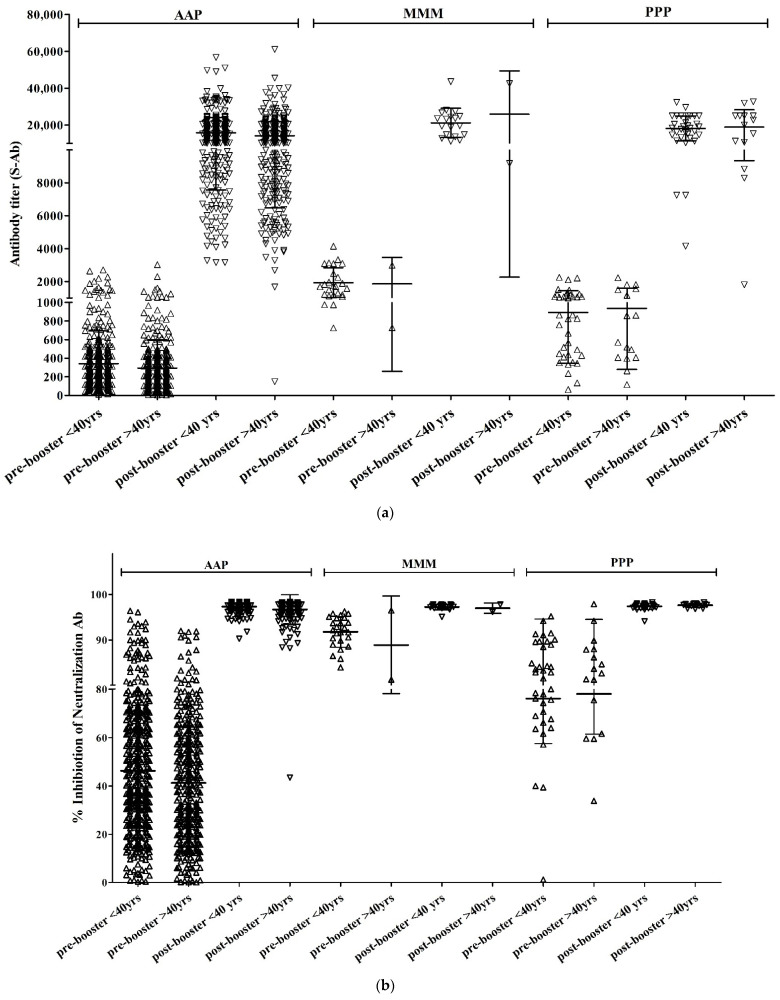
S-Ab (**a**) and Neut.Ab (**b**) titer before and after the COVID-19 booster vaccination depending on the age group.

The mean titers of S-Ab before booster vaccination were 245.8 (±2.2), 1745.3 (±1.6), and 711.9 (±2.1) in the AAP, MMM, and PPP groups, respectively, showing a statistically significant difference (*p* < 0.001) (Figure 2). All 390 participants tested positive for S-Ab after booster vaccination. The mean titer of post-booster S-Ab for each group was 13,942.5 (±1.6), 19,952.3 (±1.4), and 16,762.5 (±1.6) in the AAP, MMM, and PPP groups, respectively, showing a statistically significant difference (*p* = 0.003). There was a significant difference between the MMM and AAP groups (*p* = 0.009), but not between the AAP and PPP groups (*p* = 0.113) or between the MMM and PPP groups (*p* = 0.460).

A total of 390 participants (75.9%) tested positive for Neut.Ab before the booster vaccination. There were 329 participants (72.8%) in the AAP group, 25 (100%) in the MMM group, and 36 (97.3%) in the PPP group. Neut.Ab was positive in all participants tested after the booster vaccination. The titer of Neut.Ab before booster vaccination was significantly different depending on the prime-vaccination group; however, there was no significant difference after booster vaccination (Table 2).

A total of 396 participants (43.9%) were aged >40 years, with 107 men (27.0%) and 289 females (73.0%). There were 378, 2, and 16 participants in the AAP, MMM, and PPP groups, respectively.

The mean titers of S-Ab before booster vaccination were 204.5 (±2.4), 1475.6 (±2.7), and 712.3 (±2.3) in the AAP, MMM, and PPP groups, respectively, showing a statistically significant difference (*p* < 0.001). Post-booster S-Ab were positive in all 350 participants tested. The mean titers of post-booster S-Ab were 12,303.0 (±1.8), 19,743.9 (±3.0), and 15,473.8 (±2.2) in the AAP, MMM, and PPP groups, respectively, with no statistically significant difference (*p* = 0.203) (Table 2).

Neut.Ab was positive in 249 participants (62.9%) before the booster vaccination. There were 231 participants (61.1%) in the AAP group, 2 participants (100%) in the MMM group, and 16 participants (100%) in the PPP group. Neut.Ab was positive in all participants tested after the booster vaccination. The titer of Neut.Ab before booster vaccination was significantly different depending on the vaccination group; however, there was no significant intergroup difference after booster vaccination (Table 2).

In the AAP group, the S-Ab titer and % inhibition of Neut.Ab before and after booster vaccination were significantly higher in participants aged <40 years than those in aged >40 years. However, there were no age-dependent differences in the other vaccinated groups (Table 3).

## 4. Discussion

We evaluated the efficacy of a booster vaccination of the COVID-19 vaccine administered to those who had previously received vaccination with different types of COVID-19 vaccines. Antibody titers before booster vaccination were significantly lower in those who had previously received ChadOx1 vaccines; however, regardless of the type of primary vaccination, antibody titers after booster vaccination showed a sufficient increase. Because this study was conducted with healthcare personnel, there were no elderly participants. Accordingly, the antibody titer before booster vaccination was slightly lower in participants aged ≥40 years who received the ChadOx1 vaccine, but the effect of the booster vaccination was sufficient in this group as well as in those aged <40 years.

Various studies have demonstrated the waning immunity against COVID-19 after primary vaccination [12,13,14]. Numerous studies have been conducted on booster vaccinations against SARS-CoV-2. Most studies have been conducted using BNT162b2. There were a few studies in which the analysis was performed according to the type of vaccine previously used. The effectiveness of booster vaccination with BNT162b2 administered to participants who previously received two doses of BNT162b2 and who were >60 years of age was confirmed in a study conducted by Yinon MB et al. [15]. Later, Edson et al. reported that COVID-19 infection occurring 7 days after booster vaccination was reduced compared with placebo vaccination in participants who received a third dose of vaccination with BNT162b2 after receiving two doses of BNT162b2 [16]. Atmar et al. evaluated the effects of booster vaccinations with mRNA-1273, AD26.COV2.S, and BNT162b2 vaccines, respectively [17]. All participants showed an acceptable level of antibody escalation, for both homologous and heterologous boosters [17]. The effectiveness of the booster vaccination with BNT162b was later verified using large-scale data [18]. In general, the third-dose vaccine induces a robust response, regardless of the type of vaccine [19]. In our study, the analysis was performed according to the type of vaccine used for previous vaccinations and it was confirmed that the effect of the booster vaccination was robust.

In our study, the effect of age was investigated. Forty years is a relatively young age, but there was a case where the vaccination was stopped owing to an increase in the incidence of side effects in participants aged <40 years with the ChadOx1 vaccine at the beginning of the COVID-19 vaccination program. Accordingly, our analysis was conducted based on the age of 40 years, under the assumption that there may be differences in reactivity to vaccines. As a result, participants aged <40 years showed higher pre- and post-booster antibody titers than those aged >40 years in the AAP group. Age-dependent differences were not observed between the MMM and PPP groups.

The period of determination and implementation of the booster vaccination was from October to December 2021. As in other countries, this period was not the beginning of the spread of the omicron variant of concern. However, after booster vaccination was completed, a pandemic caused by the omicron variant occurred in South Korea from January to April 2022. Therefore, it is necessary to re-evaluate whether the third booster vaccination just before the outbreak of omicron had an effect on the omicron pandemic. However, previous studies have evaluated the effects of booster vaccination on omicron variants and subsequent subvariants at the laboratory level. If the booster vaccination was effective for omicron, it is expected that this can be similarly applied to our study [20,21,22]. In addition, although novel multivalent vaccines that cover omicron variants are continuously being developed and the efficacy of previously developed vaccines may decrease, data on booster vaccinations are still considered important for establishing healthcare policies.

Our study had several limitations. First, data on the safety and reactogenicity of the vaccinated individuals were not collected. However, the data on the safety and reactogenicity of COVID-19 vaccinations were sufficiently known from previous clinical trials. Second, the duration from the second vaccination to the booster vaccination was different in the PPP and AAP groups because the two groups received the vaccination in different periods. Therefore, it is possible that a longer duration may have affected the prevaccination antibody titer. However, as there was no difference in the post-vaccination antibody titer, the main result of the study, the difference in duration was not expected to affect the efficacy of booster vaccinations. Third, the underlying medical history of the participants was not considered in the analysis, which could influence immunogenicity [23]. Because the participants in this study were HCP, we assumed that most participants were healthy and that severely immunocompromised personnel were not included in the study or only a small proportion of these individuals were included. Furthermore, autoimmune disease status or endemic coronavirus infection status was not considered in the analysis. However, the Roche Elecsys Anti-SARS-CoV-2 S and R-find SARS-CoV-2 Neutralizing Antibody ELISA revealed 100% specificity against the anti-mitochondrial antibody- and anti-nuclear antibody-positive samples; samples from patients with rheumatoid arthritis and SLE; and coronavirus HKU1-s, NL63-, 229E-, and OC43-positive samples [24,25]. Therefore, the possibility of cross-reactivity of the anti-S antibody and neutralizing antibody tests with the above-mentioned diseases is considered to be low. Fourth, because this is not a randomized controlled trial, the age distribution varied among the groups. This raises a reasonable suspicion that the older age of the participants in the AAP group may have contributed to the lower immunogenicity. However, the trend toward lower immunogenicity in the AAP group persisted even when the participants were divided into those younger than 40 years and those older than 40 years. Therefore, it is difficult to conclude that advancing age is responsible for the lower antibody response in the AAP group. Fifth, the avidity test was not performed. Previous reports have shown that avidity increases after the second dose of vaccine, regardless of the type of vaccine. Avidity has also been reported to increase after the third dose of BNT162b2 vaccine. However, whether there is a significant difference in avidity based on the heterologous or homologous vaccine type, especially after the third vaccine dose, remains unclear and needs to be clarified [23,26,27,28,29,30].

## 5. Conclusions

Booster vaccination shows a significant effect after two doses, regardless of the type of vaccine.

## Figures and Tables

**Table 1:** Antibody titer of each vaccination group before and after the booster vaccination.

Variables	AAP (N = 837)	MMM (N = 27)	PPP (N = 53)	*p*
Age (mean, ±SD)	39.5 (11.9)	28.0 (7.3)	35.8 (9.9)	<0.001
Sex (male, *n*, %)	153 (18.3)	6 (22.2)	13 (24.5)	0.473
**Pre-booster vaccination (*n*, %)**				
S-Ab positive	837 (100)	27 (100)	53 (100)	N.A
N-Ab positive	7 (0.8)	0	0	0.714
Neut.Ab positive	566 (67.6)	27 (100)	52 (98.1)	<0.001
**Pre-booster vaccination (mean, SD)**				
S-Ab titer	228.8 (2.4)	1723.8 (1.6)	712.0 (2.2)	<0.001
Neut.Ab titer (% of inhibition)	44.2 (23.8)	91.6 (3.9)	76.6 (17.8)	<0.001
S-Ab titer *	226.1 (2.3) †	1723.8 (1.6)	712.0 (2.2)	<0.001
Neut.Ab titer * (% of inhibition)	44.0 (23.6) †	91.6 (3.9)	76.6 (17.8)	<0.001
**Post-booster vaccination (mean, SD)**				
S-Ab titer	13,088.6 (1.7) ‡	19,932.3 (1.5) §	16,360.1 (1.7) #	<0.001
Neut.Ab titer (% of inhibition)	97.0 (2.4) ‡	97.2 (0.6) §	97.5 (0.6) #	0.400
S-Ab titer *	13,105.7 (1.7) ‡	19,932.3 (1.5) §	16,360.1 (1.7) #	<0.001
Neut.Ab titer * (% of inhibition)	97.0 (2.4) ‡	97.2 (0.6) §	97.5 (0.6) #	0.404

AAP, ChadOx1-ChadOx1-BNT162b2; MMM, mRNA-1273-mRNA-1273-mRNA-1273; PPP, BNT162b2-BNT162b2-BNT162b2; S-Ab, anti-SARS-CoV-2 spike protein antibody; N-Ab, anti-SARS-CoV-2 nucleocapsid protein antibody; Neut.Ab, SARS-CoV-2 neutralization antibody; N.A., not applicable. * Exclude N-Ab positive cases who were previously infected with COVID-19. † *n* = 830, ‡ *n* = 676, § *n* = 20, # *n* = 46.

**Table 2:** Antibody analysis results according to age group.

	AAP	MMM	PPP	*p*-Value
**Subjects with no prior COVID-19 infection (age ≤ 40)**				
No. of cases with pre-booster vaccination test	452	25	37	
No. of cases with post-booster vaccination test	340	18	32	
Pre-booster S-Ab positivity (*n*, %)	452 (100%)	25 (100%)	37 (100%)	N.A
Post-booster S-Ab positivity (*n*, %)	340 (100%)	18 (100%)	32 (100%)	N.A
Pre-booster S-Ab titer (mean, ±SD)	245.8 (±2.2)	1745.3 (±1.6)	711.9 (±2.1)	<0.001
Post-booster S-Ab titer (mean, ±SD)	13,942.5 (±1.6)	19,952.3 (±1.4)	16,762.5 (±1.6)	0.003
Pre-booster Neut.Ab positivity (*n*, %)	329 (72.8%)	25 (100%)	36 (97.3%)	<0.001
Post-booster Neut.Ab positivity (*n*, %)	340 (100%)	18 (100%)	32 (100%)	N.A
Pre-booster Neut.Ab titer * (mean, ±SD)	46.3 (±23.7)	91.8 (±3.4)	76.1 (±18.5)	<0.001
Post-booster Neut.Ab titer * (mean, ±SD)	97.3 (±0.9)	97.2 (±0.6)	97.4 (±0.7)	0.803
Post-booster N-Ab positivity (*n*, %)	1 (0.1%)	0	0	N.A
**Subjects with no prior COVID-19 infection (age > 40)**				
No. of cases with pre-booster vaccination test	378	2	16	
No. of cases with post-booster vaccination test	334	2	14	
Pre-booster S-Ab positivity (*n*, %)	378 (100%)	2 (100%)	16 (100%)	N.A
Post-booster S-Ab positivity (*n*, %)	334 (100%)	2 (100%)	14 (100%)	N.A
Pre-booster S-Ab titer (mean, ±SD)	204.5 (±2.4)	1475.6 (±2.7)	712.3 (±2.3)	<0.001
Post-booster S-Ab titer (mean, ±SD)	12,303.0 (±1.8)	19,743.9 (±3.0)	15,473.8 (±2.2)	0.203
Pre-booster Neut.Ab positivity (*n*, %)	231 (61.1%)	2 (100%)	16 (100%)	0.004
Post-booster Neut.Ab positivity (*n*, %)	334 (100%)	2 (100%)	14 (100%)	N.A
Pre-booster Neut.Ab titer * (mean, ±SD)	41.2 (±23.1)	88.9 (±10.7)	78.0 (±16.5)	<0.001
Post-booster Neut.Ab titer * (mean, ±SD)	96.7 (±3.2)	97.0 (±1.1)	97.6 (±0.5)	0.566
Post-booster N-Ab positivity (*n*, %)	0	0	0	N.A

* % of inhibition.

**Table 3:** Antibody response according to vaccination group and age group.

	Age 40 or Less	Age over 40	*p*-Value
AAP			
Pre-booster S-Ab titer (mean, ±SD)	245.8 (±2.2)	204.5 (±2.4)	0.002
Post-booster S-Ab titer (mean, ±SD)	13,942.5 (±1.6)	12,303.0 (±1.8)	0.003
Pre-booster Neut.Ab (mean, ±SD), (% of inhibition)	46.3 (±23.7)	41.2 (±23.1)	0.002
Post-booster Neut.Ab (mean, ±SD), (% of inhibition)	97.3 (±0.9)	96.7 (±3.2)	0.001
MMM			
Pre-booster S-Ab titer (mean, ±SD)	1745.3 (±1.6)	1475.6 (±2.7)	0.852
Post-booster S-Ab titer (mean, ±SD)	19,952.3 (±1.4)	19,743.9 (±3.0)	0.974
Pre-booster Neut.Ab (mean, ±SD), (% of inhibition)	91.8 (±3.4)	88.9 (±10.7)	1.00
Post-booster Neut.Ab (mean, ±SD), (% of inhibition)	97.2 (±0.6)	97.0 (±1.1)	0.947
PPP			
Pre-booster S-Ab titer (mean, ±SD)	711.9 (±2.1)	712.3 (±2.3)	0.998
Post-booster S-Ab titer (mean, ±SD)	16,762.5 (±1.6)	15,473.8 (±2.2)	0.659
Pre-booster Neut.Ab (mean, ±SD), (% of inhibition)	76.1 (±18.5)	78.0 (±16.5)	0.722
Post-booster Neut.Ab (mean, ±SD), (% of inhibition)	97.4 (±0.7)	97.6 (±0.5)	0.277

## Data Availability

The data presented in this study are available upon request from the corresponding author.
